# PW01-013 – Localization of alternative pyrin isoforms

**DOI:** 10.1186/1546-0096-11-S1-A66

**Published:** 2013-11-08

**Authors:** E Tahir Turanli, S Erdemir, G Celikyapi Erdem

**Affiliations:** 1 Department of Molecular Biology and Genetics , Istanbul Technical University, Turkey; 2 Molecular Biology-Biotechnology and Genetics Research Center, Istanbul Technical University, Istanbul, Turkey

## Introduction

The importance of MEFV gene protein, Pyrin/Marenostrin (P/M) in the inflammatory pathway is well established. P/M is expressed in neutrophils, eosinophils, monocytes, dendritic cells and synovial fibroblasts. There are many MEFV transcripts which are generated by alternative splicing events including deleted exons 2,3,4,5,7 and 8 in several combinations or individually. Some of these transcripts (2a, 2d, 8ext, 2d/8ext and 2d/9ext) were shown to be expressed as protein isoforms in leukocytes. A previous study carried out by our group has shown that exon 2 deleted form (d2) in leukocyte samples of FMF patients is expressed more than 400 fold compared to healthy control samples (p=0.026).

## Objectives

Based on the hypothesis that different localizations and functions for full length and MEFV alternatively spliced transcripts, this study aimed to determine the localization differences between full-length P/M and P/M-d2 protein isoforms in neutrophil-like cells *in vitro*.

## Methods

Two GFP-tagged plasmids which are are pCMV6-AC-GFP + MEFV-fl (MEFV-fl-GFP) and pCMV6-AC-GFP + MEFV-d2 (MEFV-d2-GFP) were transfected to HL-60 (Human promyelocytic leukemia cells) cell lines and examined via confocal microscopy. Subsequently, six-day incubation with 1.75% DMSO was performed to differentiate HL-60 cells to neutrophil-like cells. These cells were also transfected with same plasmids and proteins were observed through confocal microscopy technique.

## Results

Transfection studies showed that MEFV-fl-GFP was cytoplasmic and MEFV-d2-GFP was nuclear in HL-60 cell line. On the other hand, both MEFV-fl-GFP and MEFV-d2-GFP were localized in cytoplasm of neutrophil-like cells.

## Conclusion

In previous studies, cellular localization of P/M-fl and P/M-d2 was investigated in several cell lines through using transfection methods or P/M antibody. Transfection studies showed that full-length P/M was generally cytoplasmic and 2Δ isoform was the only isoform which can enter nucleus but may also localize in cytoplasm. However localization studies using P/M antibodies, which cannot currently distinguish between different isoforms, showed that although native P/M consists of predominantly full-length type, protein was also observed in the nucleus of neutrophils. Our localization results of P/M-fl and P/M-d2 in HL-60 cells were compatible with literature, they were observed in the cytoplasm and nucleus, respectively. On the other hand, both P/M-d2 and P/M-fl isoforms were found to be localized only in cytoplasm not in nucleus in the neutrophil-like cells. These findings had led us to suggest that post-transcriptional modifications for P/M-d2 that may occur during cell differentiation or possibly through inflammation such that its natural localization in the nucleus may point to its role in the inflammation maybe like a transcription factor.

## Disclosure of interest

None declared

